# Digestible lysine requirements of male broilers from 1 to 42 days of age reassessed

**DOI:** 10.1371/journal.pone.0179665

**Published:** 2017-06-21

**Authors:** Henrique Scher Cemin, Sergio Luiz Vieira, Catarina Stefanello, Marcos Kipper, Liris Kindlein, Ariane Helmbrecht

**Affiliations:** 1 Department of Animal Science, Federal University of Rio Grande do Sul, Porto Alegre, Rio Grande do Sul, Brazil; 2 Department of Preventive Veterinary Medicine, Federal University of Rio Grande do Sul, Porto Alegre, Rio Grande do Sul, Brazil; 3 Evonik Nutrition & Care GmbH, Hanau-Wolfgang, Germany; Leibniz-Institut fur Pflanzengenetik und Kulturpflanzenforschung Gatersleben, GERMANY

## Abstract

Three experiments were conducted separately to estimate the digestible Lys (dig. Lys) requirements of Cobb × Cobb 500 male broilers using different statistical models. For each experiment, 1,200 chicks were housed in 48 floor pens in a completely randomized design with 6 treatments and 8 replicates. Broilers were fed diets with increasing dig. Lys levels from 1 to 12 d (Exp. 1), from 12 to 28 d (Exp. 2), and 28 to 42 d (Exp. 3). Increasing dig. Lys levels were equally spaced from 0.97 to 1.37% in Exp. 1, 0.77 to 1.17% in Exp. 2, and 0.68 to 1.07% in Exp. 3. The lowest dig. Lys diets were not supplemented with L-Lysine and all other essential AA met or exceeded recommendations. In Exp. 3, six birds per pen were randomly selected from each replication to evaluate carcass and breast yields. Digestible Lys requirements were estimated by quadratic polynomial (QP), linear broken-line (LBL), quadratic broken-line (QBL), and exponential asymptotic (EA) models. Overall, dig. Lys requirements varied among response variables and statistical models. Increasing dietary dig. Lys had a positive effect on BW, carcass and breast yields. Levels of dig. Lys that optimized performance using QP, LBL, QBL, and EA models were 1.207, 1.036, 1.113, and 1.204% for BWG and 1.190, 1.027, 1.100, and 1.172% for FCR in Exp. 1; 1.019, 0.853, 0.944; 1.025% for BWG and 1.050, 0.879, 1.032, and 1.167% for FCR in Exp. 2; and 0.960, 0.835, 0.933, and 1.077% for BWG, 0.981, 0.857, 0.963, and 1.146% for FCR in Exp. 3. The QP, LBL, QBL, and EA also estimated dig. Lys requirements as 0.941, 0.846, 0.925, and 1.070% for breast meat yield in Exp. 3. In conclusion, Lys requirements vary greatly according to the statistical analysis utilized; therefore, the origin of requirement estimation must be taken into account in order to allow adequate comparisons between references.

## Introduction

Lysine (Lys) is the second limiting amino acid (AA) in practical corn-soybean meal diets. Growth performance and proper muscle development of broiler chickens is highly dependent of an adequate dietary supply of Lys. Although all AA coded in the nucleic acid sequence are required for muscle protein synthesis, also depending on its frequency and dietary supply, Lys is the main essential AA required for muscle building for broilers [1]. Lysine has also gained importance in the expression of essential AA requirements as its ratio, which is the basis of the popularly known ideal protein concept [2]. Estimation of accurate Lys requirements can greatly affect broiler performance and meat yields because minor deviations lead to the inadequate inclusions of all other indispensable AA [3].

Nutrient recommendations for broilers intend to optimize growth performance as well as the proportions of individual carcass components, especially breast meat, to a point that best economic returns are obtained. It has been reported that Lys can increase carcass yields and alter its composition by increasing meat yield and reducing carcass fat [1,4]. This trend has been exacerbated in the last years since breast muscles, and therefore, meat yields, have been steadily grown as a proportion of the other body components of broiler chickens.

Dietary Lys also plays an important role in breast muscle protein turnover by modulating protein synthesis and breakdown rates [5]. Furthermore, Lys deficiency results in reduced protein mass, especially in the *Pectoralis major*, which is more sensitive to Lys than wings and thigh muscles [6]. The breast muscles are particularly sensitive to dietary concentration of Lys since it represents approximately 7% of the total body protein content [7]. In contrast to leg muscles, breast muscles are a direct product of genetic selection, have minor functional purpose, and represent a considerable protein storage during periods of nutrient deficiency [6].

Usual determinations of AA requirements target the optimization of growth performance, feed conversion ratio (FCR), and breast meat yields; however, AA concentrations that optimize breast meat yields have shown to be higher than for the other responses [8,9,10]. Improvements in broiler growth performance could indicate a greater AA requirement than determined with previous researches. The last edition of the NRC (1994) [11] is obviously outdated now, but just as a matter of comparison, it suggests 1.10% total Lys from 0 to 21 d and 1.00% total Lys from 21 to 42 d. Recent research [12–14] has demonstrated that the Lys requirements of modern broilers are higher than those previously recommended, although many factors such as strain, sex, age, type of diet, levels of other nutrients, environment, response criteria, and statistical model are capable of influencing the requirements obtained from different studies [3].

Diverse regression methods have been used to estimate Lys requirements for broilers. Pesti et al. (2009) [15] tested different statistical models using the same data set and observed that Lys requirement estimates ranged from 0.90% to 1.28% depending on the model used (quadratic broken-line, quadratic polynomial, linear broken-line, and exponential asymptotic). Other authors recently presented digestible Lys (dig. Lys) requirements from 1 to 14 d using the quadratic broken-line model (QBL) as 1.180% for body weight gain (BWG) and 1.261% for FCR [14], 1.07% for BWG and 1.10% for FCR, respectively, using quadratic polynomial (QP) regression, and as 1.09% for BWG and 1.15% for FCR, respectively, based on QBL model [16] from 14 to 28 d. An older study [12], determined requirements for BWG as 1.13% total Lys from 14 to 28 d and 0.94% for BWG and 0.95% for FCR from 28 to 42 d using linear broken-line (LBL) model.

An adequate statistical model is essential to correctly interpret the requirements [12,14,17–19]. Multiple-range tests are not suitable to estimate requirements because their outcome is between two tested concentrations of the AA, hence no precise requirement prediction is possible [15,20]. There are many models that relate performance variables with feed composition, such as QP, LBL, QBL, and exponential asymptotic (EA), which have been thoroughly reviewed by Pesti et al. (2009) [15] and were used in the present study. The objective of the present research was to estimate the dig. Lys requirements of Cobb x Cobb 500 male broilers from 1 to 12 d, 12 to 28 d, and 28 to 42 d-of-age using different statistical regression models such that further comparisons regarding this nutrient can be performed.

## Materials and methods

All procedures utilized in the present study were approved by the Ethics and Research Committee of the Federal University of Rio Grande do Sul, Porto Alegre, Brazil. The Ethics Committee approved this study (approval number 216/2015).

### Bird husbandry

Three experiments (Exp.) were conducted using 1,200 one-d-old male, slow-feathering Cobb 500 x Cobb broilers. Chicks were vaccinated for Marek’s and infectious bursal diseases at the hatchery (BRF, Lajeado, Brazil) and then randomly distributed into 48 pens of 1.65 × 1.65 m (9.2 birds/m^2^, 25 birds per pen). Experimental pens had rice hulls as bedding, one 15 kg capacity tube feeder, and 3 nipple drinkers. Mash feeds and water were available for *ad libitum* consumption. Mortality was recorded daily. Initial temperature was set to 32°C being reduced to maintain bird comfort throughout the studies. A continuous lighting schedule was used until 7 d-of-age, whereas a 20L:4D cycle with constant intensity was used thereafter.

Experiments 1, 2, and 3 were designed to estimate Lys requirement from 1 to 12 d, 12 to 28 d, and 28 to 42 d-of-age, respectively. In Exp. 1, birds were fed the experimental diets from placement to 12 d whereas in Exp. 2 and 3, birds received common standard commercial diets based on corn and soybean meal until the start of the experimental period (Table 1). The standard common diets from 1 to 12, 12 to 28, and 28 to 42 d had 24.2, 21.4, and 20.0% CP; 1.05, 0.80, and 0.66% Ca, and 0.52, 0.40, and 0.33% Av. P, respectively (Table 2). Birds and feeds were weighed on d 1 and 12 in Exp. 1, d 12 and 28 in Exp. 2, and d 28 and 42 in Exp. 3 when BWG, feed intake (FI), and FCR corrected for the weight of dead birds were calculated.

**Table 1:** Ingredient and nutrient composition of experimental diets.

Item	Experiment 1	Experiment 2	Experiment 3
Ingredient, %			
Corn 7.8% CP	57.31	67.97	75.54
Soybean meal 45% CP	30.34	21.54	14.32
Corn gluten meal	5.00	5.50	5.80
Soybean oil	2.49	0.94	0.80
Dicalcium phosphate	1.39	0.94	0.52
Limestone	1.34	1.06	0.91
Salt	0.29	0.14	0.05
Sodium bicarbonate	0.32	0.39	0.57
DL-Met 99%	0.32	0.26	0.27
L-Thr 98.5%	0.14	0.14	0.09
L-Arg 98%	0.12	0.14	0.20
L-Ile 98.5%	0.08	0.08	0.06
L-Val 96.5%	0.13	0.11	0.07
L-Trp 98%	-	0.01	0.01
L-Leu 98.5%	-	0.03	-
Vitamin and mineral mix^1^	0.15	0.15	0.15
Choline chloride 60%	0.08	0.10	0.14
Kaolin	0.50	0.50	0.50
Nutritional composition, % or as noted^2^			
AME_n_, kcal/kg	3,035	3,108	3,180
Crude protein	22.82 (22.45)	19.48 (19.90)	18.85 (19.00)
Ca, %	1.05	0.84	0.68
Available P, %	0.52	0.42	0.33
Na, %	0.24	0.19	0.20
DEB^3^, mEq/kg	220	190	180
Choline, mg/kg	1,600	1,550	1,500
dig. TSAA	0.97 (1.02)	0.83 (0.87)	0.80 (0.81)
dig. Lys	0.97 (1.12)	0.77 (0.85)	0.68 (0.77)
dig. Thr	0.84 (0.97)	0.73 (0.81)	0.72 (0.79)
dig. Val	1.05 (1.17)	0.89 (0.98)	0.87 (1.03)
dig. Ile	0.92 (1.03)	0.78 (0.82)	0.74 (0.75)
dig. Leu	1.97 (2.20)	1.79 (1.91)	1.77 (1.89)
dig. Arg	1.38 (1.52)	1.16 (1.25)	1.12 (1.24)

^1^ Composition per kg of feed: vit. A, 8,000 UI; vit. D3, 2,000 UI; vit. E, 30 UI; vit. K3, 2 mg; thiamine, 2 mg; riboflavin, 6 mg; pyridoxine, 2.5 mg; cyanocobalamine, 0.012 mg, panthothenic acid, 15 mg; niacin, 35 mg; folic acid, 1 mg; biotin; iron, 40 mg; zinc, 80 mg; manganese, 80 mg; copper, 10 mg; iodine, 0.7 mg; selenium, 0.3 mg; phytase, 100 mg; monensin sodium, 100 mg.

^2^ Values in parenthesis are analyzed and AA digestibility followed Rostagno et al. (2011) [21].

^3^ Dietary electrolyte balance represents dietary Na + K–Cl in mEq/kg of diet.

**Table 2:** Ingredient and nutrient composition of the common diets^1^.

Item	Starter	Grower	Finisher
Ingredients, %			
Corn	47.48	54.73	59.39
Soybean meal	44.45	36.98	33.07
Soybean oil	4.04	5.33	5.11
Sodium bicarbonate	0.08	0.01	0.02
Dicalcium phosphate	1.33	0.74	0.40
Limestone	1.35	1.03	0.85
Salt	0.50	0.45	0.42
Vitamin and mineral mix^2^	0.15	0.15	0.15
DL-Methionine, 99%	0.40	0.34	0.31
L-Lysine HCl, 78%	0.14	0.16	0.18
L-Threonine, 98.5%	0.05	0.04	0.04
Choline chloride, 60%	0.03	0.04	0.06
Energy and nutrients, % or unless noted			
AME_n_, kcal/kg	2,960	3,150	3,200
Crude protein	24.20	21.40	20.00
Ca	1.05	0.80	0.66
Av. P	0.52	0.40	0.33
Choline, mg/kg	1,600	1,500	1,500
dig. Lys	1.34	1.18	1.10
dig. Met + Cys	1.03	0.91	0.85
dig. Thr	0.87	0.77	0.72
dig. Val	1.03	0.91	0.85

^1^ Exp. 1: grower and finisher provided after the experimental phase (1 to 12 d); Exp. 2: starter and finisher provided before and after the experimental phase (12 to 28 d), respectively; Exp. 3: starter and grower provided before the experimental phase (28 to 42 d).

^2^ Composition per kg of feed: vit. A, 8,000 UI; vit. D_3_, 2,000 UI; vit. E, 30 UI; vit. K_3_, 2 mg; thiamine, 2 mg; riboflavin, 6 mg; pyridoxine, 2.5 mg; cyanocobalamine, 0.012 mg, panthothenic acid, 15 mg; niacin, 35 mg; folic acid, 1 mg; biotin, 0.08 mg; iron, 40 mg; zinc, 80 mg; manganese, 80 mg; copper, 10 mg; iodine, 0.7 mg; selenium, 0.3 mg; phytase, 100 mg, monensin sodium, 100 mg.

In the Exp. 3, six birds per pen (n = 288) were selected within the upper and lower 5% of each pen average at 43 d of age for processing to carcass and breast yields evaluation. Birds were fasted for 6 h, individually weighed before electrical stunning (45 V for 3 s), bled for 3 min after carotid and jugular veins cut, scalded at 60°C for 45 s, and mechanically defeathered. Carcasses were manually eviscerated and then statically chilled in slush ice for 3 h before processing. Breast fillets were manually deboned from the carcasses. Carcass yield was expressed as a percentage of live weight and breast yield was expressed as a percentage of the eviscerated carcass weight.

### Experimental diets

Dietary treatments in Exp. 1 were provided from 1 to 12 d-of-age, in Exp. 2 from 12 to 28 d-of-age and in Exp. 3 from 28 to 42 d-of-age. Diets in the experiments were based on corn, soybean meal, and corn gluten meal [21]. Basal diets were formulated without supplemental L-Lysine (L-Lys) (0.97% dig. Lys in Exp. 1, 0.77% dig. Lys in Exp. 2 and 0.68% of dig. Lys in Exp. 3, respectively), and contained all other essential AA that meet or exceed commercial recommendations to ensure dietary adequacy such that responses were only limited by dig. Lys.

Treatments were structured with the addition of increasing levels of dig. Lys in 0.08% increments from 0.97% to 1.37% in Exp. 1, from 0.77 to 1.17% in Exp. 2, and from 0.68 to 1.07% in Exp. 3 by adding L-Lysine HCl at the expense of an inert filler (kaolin). Standard corn-SBM-based broiler diets were provided to all treatments in the periods after (grower diet grower from 12 to 28 d-of-age; finisher from 28 to 42 d-of-age) the experimental phase of Exp. 1, before (starter diet from 1 to 12 d-of-age) and after (finisher diet from 28 to 35 d-of-age) experimental phases of Exp. 2, and before (starter diet from 1 to 12 d-of-age; grower from 12 to 28 d-of-age) the experimental phase of Exp. 3 (Table 2). Before diets formulation, near infrared reflectance spectroscopy was used to analyze the ingredients for crude protein (CP) and AA contents [22,23] and analyzed values were used to determine diet formulation applying digestibility coefficients [24] to analyzed total AA content. Diets were manufactured in mash form and representative samples of each complete feed were obtained to validate Lys concentrations.

### Statistical analysis

The experiments had a gradient treatment structure in a completely randomized design. Treatments had 8 replications in every experiment. Four mathematical models were used to estimate the optimal level of dig. Lys inclusion to maximize broiler responses based on a gradient treatment structure [15,25–27]. Body weight gain, FCR, and carcass traits were selected as broiler responses. Linear broken-line, QBL, and EA models were estimated using a nonlinear procedure, whereas the QP regression model was estimated using a regression procedure [28]. Models were summarized as follows: the QP regression model was expressed as Y = β_0_ + *β*_1_ × X + *β*_2_ × X_2_, where Y is the dependent variable, X is the dietary Lys concentration, and *β*_*0*_ is the intercept, *β*_1_ and *β*_2_ are the linear and quadratic coefficients, respectively; maximum response concentration was obtained by:—*β*_1_ ÷ (2 × *β*_2_). The LBL model was expressed as Y = β_0_ + *β*_1_ × (*β*2—X), where (*β*_2_—X) = 0 for X > *β*_2_, Y is the dependent variable, X is the dietary Lys concentration, *β*_0_ is the value at the plateau, *β*_1_ is the slope and *β*_2_ is the Lys concentration at the break point. Quadratic broken-line model is Y = *β*_0_ + *β*_1_ × (*β*_2_—X)^2^, where (*β*_2_—X) = 0 for X > *β*_2_, Y is the dependent variable, X is the dietary Lys concentration, *β*_0_ is the value at the plateau, *β*_1_ is the slope and *β*_2_ is the Lys concentration at the break point. The EA was expressed as Y = *β*_0_ + *β*_1_ × (1 –EXP(– *β*_2_ × (X– *β*_3_), where Y is the dependent variable, X is the dietary Lys concentration, *β*_0_ is the response for the dependent variable estimated for the feed with the lower Lys, *β*_1_ is the difference estimated between the minimum and maximum response obtained by the increasing Lys, *β*_2_ is the slope of the exponential curve, *β*_3_ is the Lys at the lower level; requirement were estimated by calculating (ln(0.05) *÷ – β*_2_) + *β*_3_ for 95% of the requirement and (ln(0.01) *÷ – β*_2_) + *β*_3_ for 99%.

## Results

Overall, there were no statistical differences between % mortality in any of the experiments (*P* > 0.05). Overal means for the period mortality in the 3 studies were 0.37%, 0.64% and 0.52% in Exp. 1, 2, and 3, respectively.

### Experiment 1–1 to 12 d of age

Feeding Cobb × Cobb 500 birds with increasing levels of dig. Lys resulted in a positive linear response (*P* ≤ 0.01) for dig. Lys intake and quadratic responses (*P* ≤ 0.01) for BWG and FCR (Table 3). Digestible Lys requirements estimates based on 100 and 99% of optimal responses were calculated and are shown in Table 4. The QP regression equations estimated 95% of dig. Lys requirement at 1.207% and 1.190% for BWG and FCR, respectively; the LBL model estimates were the lowest at 1.036% and 1.027% for BWG and FCR, respectively; the QBL model estimated at 1.113% for BWG and 1.100% for FCR; and the EA model estimated as 1.204% for BWG and 1.172% for FCR. When averaged across variables, 95% of dig. Lys requirement was estimated as 1.199% using the QP regression, 1.032% using LBL model, 1.107% using QBL model, and 1.188% using the EA model. The best fit was provided by the QBL for both BWG and FCR since its R^2^ was the highest (0.88 for BWG and 0.74 for FCR).

**Table 3:** Growth performance of broilers fed gradient levels of dig. Lys from 1 to 12 d of age^1^ (Experiment 1).

Item	BW gain, g	FCR^2^, g/g	Feed intake, g	Lys intake, g/d
Digestible Lys, %				
0.97%	295.4	1.377	406.8	0.329
1.05%	340.8	1.241	423.0	0.370
1.13%	359.7	1.206	433.5	0.408
1.21%	361.2	1.181	426.3	0.430
1.29%	366.5	1.178	431.7	0.464
1.37%	367.1	1.192	437.5	0.500
SEM	3.92	0.012	2.69	0.008
*P*-value				
Linear response	0.0001	0.0001	0.1332	0.0001
Quadratic response	0.0001	0.0001	0.1819	0.3285

^1^ Values are least square means of 8 replicates with 25 birds each for 0.97% digestible Lys and 16 replicates with 25 birds each for all other treatments.

^2^ Feed conversion ratio corrected for mortality weight.

**Table 4:** Digestible Lys requirements from 1 to 12 d of age (Experiment 1).

Model	Response variable	Equation	Estimated requirement (100, 99, 95%)	*P*-value	R^2^
Quadratic polynomial^1^	BW gain	y = -882.2 + 1972.3x – 776.2x^2^	1.271, 1.258, 1.207	0.0001	0.8404
	FCR^5^	y = 5.022–6.156x + 2.457x^2^	1.253, 1.240, 1.190	0.0001	0.7135
Linear broken-line^2^	BW gain	y = 363.6–568.0 × (1.090 –x)	1.090, 1.079, 1.036	0.0001	0.8732
	FCR	y = 1.189 + 1.696 × (1.081 –x)	1.081, 1.070, 1.027	0.0001	0.7428
Quadratic broken-line^3^	BW gain	y = 364.6–1685.8 × (1.172 –x)^2^	1.172, 1.160, 1.113	0.0001	0.8765
	FCR	y = 1.187 + 5.315 × (1.158 –x)^2^	1.158, 1.146, 1.100	0.0001	0.7441
Exponential asymptotic^4^	BW gain	Y = 261 + 106 × (1 –EXP(– 12.829 × (X– 0.97)))	–, 1.329, 1.204	0.0001	0.8743
	FCR	Y = 1.524–342 × (1 –EXP(– 14.825 × (X– 0.97)))	–, 1.281, 1.172	0.0001	0.7437

^1^ Quadratic polynomial regression model (QP): Y = *β*_0_ + *β*_1_ × X + *β*_2_ × X^2^, where Y is the dependent variable, X is the dietary Lys concentration, and *β*_*0*_ is the intercept, *β*_1_ and *β*_2_ are the linear and quadratic coefficients, respectively; maximum response concentration was obtained by:—*β*_1_ ÷ (2 × *β*_2_).

^2^ Linear broken-line model (LBL): Y = *β*_0_ + *β*_1_ × (*β*_2_—X), where (*β*_2_—X) = 0 for X > *β*_2_, Y is the dependent variable, X is the dietary Lys concentration, *β*_0_ is the value at the plateau, *β*_1_ is the slope and *β*_2_ is the Lys concentration at the break point.

^3^ Quadratic broken-line model (QBL): Y = *β*_0_ + *β*_1_ × (*β*_2_—X)^2^, where (*β*_2_—X) = 0 for X > *β*_2_, Y is the dependent variable, X is the dietary Lys concentration, *β*_0_ is the value at the plateau, *β*_1_ is the slope and *β*_2_ is the Lys concentration at the break point.

^4^ Exponential asymptotic (EA): Y = *β*_0_ + *β*_1_ × (1 –EXP(– *β*_2_ × (X– *β*_3_), where Y is the dependent variable, X is the dietary Lys concentration, *β*_0_ is the response for the dependent variable estimated for the feed with the lower Lys, *β*_1_ is the difference estimated between the minimum and maximum response obtained by the increasing Lys, *β*_2_ is the slope of the exponential curve, *β*_3_ is the Lys at the lower level; requirement were estimated by calculating (ln(0.05)/– *β*_2_) + *β*_3_ for 95% of the requirement and (ln(0.01)/– *β*_2_) + *β*_3_ for 99%.

^5^ Feed conversion ratio corrected for mortality weight.

### Experiment 2–12 to 28 d of age

Significant quadratic responses (*P* ≤ 0.01) were observed for BWG, FCR, feed intake, and dig. Lys intake in the period from 12 to 28 d (Table 5). Digestible Lys requirements estimates based on 100, 99%, and 95% of optimal responses were calculated and are presented in Table 6. Digestible Lys requirements were estimated at 1.019% for BWG and 1.050% for FCR using the QP regression whereas the LBL model estimates were 0.853% for BWG and 0.879% for FCR. As in Exp. 1, the QBL model provided the best fit, which, at 95% of maximum response were 0.944% and 1.032% for BWG and FCR, respectively. The EA model estimated dig. Lys requirements as 1.025% for BWG and 1.167% for FCR. When averaged across variables, dig. Lys requirement were estimated as 1.035% using QP regression, 0.866% using LBL model, 0.988% using QBL model, and 1.096% using the exponential model.

**Table 5:** Growth performance of broilers fed gradient levels of dig. Lys from 12 to 28 d of age^1^ (Experiment 2).

Item	BW gain, g	FCR^2^, g/g	Feed intake, g	Lys intake, g/d
Digestible Lys, %				
0.77%	1,040	1.734	1,803	1.16
0.85%	1,186	1.626	1,928	1.37
0.93%	1,255	1.564	1,964	1.52
1.01%	1,282	1.522	1,951	1.64
1.09%	1,271	1.505	1,913	1.74
1.17%	1,290	1.507	1,943	1.90
SEM	13.14	0.012	9.10	0.04
*P*-value				
Linear response	0.0001	0.0001	0.0001	0.0001
Quadratic response	0.0001	0.0001	0.0001	0.0001

^1^ Values are least square means of 8 replicates with 25 birds each for 0.77% digestible Lys and 16 replicates with 25 birds each for all other treatments.

^2^ Feed conversion ratio corrected for mortality weight.

**Table 6:** Digestible Lys requirement from 12 to 28 d of age (Experiment 2).

Model	Response variable	Equation	Estimated requirement (100, 99, 95%)	*P*-value	R^2^
Quadratic polynomial^1^	BW gain	Y = -1777.1 + 5726.0x – 2670.0x^2^	1.072, 1.062, 1.019	0.0001	0.9107
	FCR^5^	Y = 3.983–4.490x + 2.031x^2^	1.105, 1.094, 1.050	0.0001	0.9568
Linear broken-line^2^	BW gain	Y = 1274.5–1830.3 × (0.898 –x)	0.898, 0.889, 0.853	0.0001	0.9329
	FCR	Y = 1.525 + 1.352 × (0.925 –x)	0.925, 0.916, 0.879	0.0001	0.9043
Quadratic broken-line^3^	BW gain	Y = 1280.2–4757.2 × (0.994 –x)^2^	0.994, 0.984, 0.944	0.0001	0.9418
	FCR	Y = 1.507 + 2.247 × (1.086 –x)^2^	1.086, 1.075, 1.032	0.0001	0.9578
Exponential asymptotic^4^	BW gain	Y = 960 + 329 × (1 –EXP(– 11.741 × (X– 0.77)))	–, 1.162, 1.025	0.0001	0.9411
	FCR	Y = 1.809–0.321 × (1 –EXP(– 7.544 × (X– 0.77)))	–, 1.380, 1.167	0.0001	0.9560

^1^ Quadratic polynomial regression model (QP): Y = *β*_0_ + *β*_1_ × X + *β*_2_ × X^2^, where Y is the dependent variable, X is the dietary Lys concentration, and *β*_*0*_ is the intercept, *β*_1_ and *β*_2_ are the linear and quadratic coefficients, respectively; maximum response concentration was obtained by:—*β*_1_ ÷ (2 × *β*_2_).

^2^ Linear broken-line model (LBL): Y = *β*_0_ + *β*_1_ × (*β*_2_—X), where (*β*_2_—X) = 0 for X > *β*_2_, Y is the dependent variable, X is the dietary Lys concentration, *β*_0_ is the value at the plateau, *β*_1_ is the slope and *β*_2_ is the Lys concentration at the break point.

^3^ Quadratic broken-line model (QBL): Y = *β*_0_ + *β*_1_ × (*β*_2_—X)^2^, where (*β*_2_—X) = 0 for X > *β*_2_, Y is the dependent variable, X is the dietary Lys concentration, *β*_0_ is the value at the plateau, *β*_1_ is the slope and *β*_2_ is the Lys concentration at the break point.

^4^ Exponential asymptotic (EA): Y = *β*_0_ + *β*_1_ × (1 –EXP(– *β*_2_ × (X– *β*_3_), where Y is the dependent variable, X is the dietary Lys concentration, *β*_0_ is the response for the dependent variable estimated for the feed with the lower Lys, *β*_1_ is the difference estimated between the minimum and maximum response obtained by the increasing Lys, *β*_2_ is the slope of the exponential curve, *β*_3_ is the Lys at the lower level; requirement were estimated by calculating (ln(0.05)/– *β*_2_) + *β*_3_ for 95% of the requirement and (ln(0.01)/– *β*_2_) + *β*_3_ for 99%.

^5^ Feed conversion ratio corrected for mortality weight.

### Experiment 3–28 to 42 d of age

Growth performance and broiler processing data from 28 to 42 d are presented in Table 7. Providing broilers gradient concentrations of dig. Lys resulted in a linear response (*P* ≤ 0.01) for dig. Lys intake, and quadratic responses (*P* ≤ 0.05) for BWG, FCR, feed intake, dig. Lys intake, carcass yield, and breast meat yield from 28 to 42 d. Digestible Lys requirements estimates based on 100, 99%, and 95% of optimal responses were calculated and are represented in Table 8. The QP equations estimated dig. Lys requirements at 0.960, 0.981, 0.903, and 0.941% for BWG, FCR, carcass yield, and breast meat yield, respectively. The LBL model estimates were the lowest at 0.835% for BWG, 0.857% for FCR, 0.834 for carcass yield, and 0.846% for breast meat yield. The QBL model estimated dig. Lys requirements at 0.933, 0.963, 0.911, and 0.925% for BWG, FCR, carcass yield, and breast meat yield, respectively. The EA model estimated dig. Lys requirements as 1.077% for BWG, 1.146% for FCR, 1.032% for carcass yield, and 1.070% for breast meat yield. When averaged across variables, dig. Lys requirements were calculated as 0.946% using QP regression analysis, 0.843% using LBL model, 0.933% using QBL model, and 1.081% using the EA model. As in Exp. 1 and 2, QBL model provided the best fit for BWG and FCR. However, for the carcass yield, the LBL provided the best fit.

**Table 7:** Growth performance and processing yields of broilers fed gradient levels of dig. Lys from 28 to 42 d of age^1^ (Experiment 3).

Item	BW gain, g	FCR^2^, g/g	Feed intake, g	Lys intake, g/d	Carcass yield^3^, %	Breast meat yield^4^, %
Digestible Lys, %						
0.68%	1,457	2.039	2,970	1.44	78.60	21.66
0.76%	1,594	1.899	3,029	1.64	78.89	23.01
0.84%	1,709	1.809	3,090	1.85	80.07	24.44
0.92%	1,769	1.716	3,036	1.99	80.64	25.65
1.00%	1,769	1.721	3,042	2.17	80.34	25.17
1.08%	1,782	1.700	3,030	2.34	79.82	25.15
SEM	18.54	0.018	11.91	0.05	0.16	0.23
*P*-value						
Linear response	0.0001	0.0001	0.0250	0.0001	0.0006	0.0001
Quadratic response	0.0001	0.0001	0.0298	0.0706	0.0013	0.0001

^1^ Values are least square means of 8 replicates with 25 birds each for 0.68% digestible Lys and 16 replicates with 25 birds each for all other treatments.

^2^ Feed conversion ratio corrected for mortality weight.

^3^ Means from 8 replicates of 6 birds each (n = 288); carcass yield as a percentage of live weight.

^4^ Breast meat yield expressed as a percentage of carcass weight.

**Table 8:** Digestible Lys requirement from 28 to 42 d of age (Experiment 3).

Model	Response variable	Equation	Estimated requirement (100, 99, 95%)	*P*-value	R^2^
Quadratic polynomial^1^	BW gain	Y = -1295.9 + 6102.9x – 3019.3x^2^	1.011, 1.001, 0.960	0.0001	0.8632
	FCR^5^	Y = 4.605–5.629x + 2.727x^2^	1.032, 1.022, 0.981	0.0001	0.9500
	Carcass yield	Y = 55.32 + 52.72x – 27.72x^2^	0.951, 0.941, 0.903	0.0001	0.3930
	Breast meat yield	Y = -14.43 + 80.51x – 40.64x^2^	0.990, 0.981, 0.941	0.0001	0.7599
Linear broken-line^2^	BW gain	Y = 1773.4–1572.7 × (0.879 –x)	0.879, 0.870, 0.835	0.0001	0.8564
	FCR	Y = 1.712 + 1.437 × (0.902 –x)	0.902, 0.893, 0.857	0.0001	0.9417
	Carcass yield	Y = 80.27–9.13 × (0.878 –x)	0.878, 0.869, 0.834	0.0001	0.3815
	Breast meat yield	Y = 25.32–17.42 × (0.891 –x)	0.891, 0.882, 0.846	0.0001	0.7713
Quadratic broken-line^3^	BW gain	Y = 1777.3–3561.5 × (0.982 –x)^2^	0.982, 0.972, 0.933	0.0001	0.8664
	FCR	Y = 1.707 + 3.002 × (1.014 –x)^2^	1.014, 1.004, 0.963	0.0001	0.9511
	Carcass yield	Y = 80.25–23.51 × (0.959 –x)^2^	0.959, 0.949, 0.911	0.0001	0.3627
	Breast meat yield	Y = 25.30–43.83 × (0.974 –x)^2^	0.974, 0.964, 0.925	0.0001	0.7574
Exponential asymptotic^4^	BW gain	Y = 1335 + 472 × (1 –EXP(– 7.544 × (X– 0.68)))	–,1.290, 1.077	0.0001	0.8588
	FCR	Y = 2.079–0.415 × (1 –EXP(– 6.433 × (X– 0.68)))	–,1.396, 1.146	0.0001	0.9456
	Carcass yield	Y = 76.39 + 3.97 × (1 –EXP(– 8.499 × (X– 0.68)))	–,1.222, 1.032	0.0001	0.3332
	Breast meat yield	Y = 19.88 + 5.75 × (1 –EXP(– 7.687 × (X– 0.68)))	–,1.279, 1.070	0.0001	0.7344

^1^ Quadratic polynomial regression model (QP): Y = *β*_0_ + *β*_1_ × X + *β*_2_ × X^2^, where Y is the dependent variable, X is the dietary Lys concentration, and *β*_*0*_ is the intercept, *β*_1_ and *β*_2_ are the linear and quadratic coefficients, respectively; maximum response concentration was obtained by:—*β*_1_ ÷ (2 × *β*_2_).

^2^ Linear broken-line model (LBL): Y = *β*_0_ + *β*_1_ × (*β*_2_—X), where (*β*_2_—X) = 0 for X > *β*_2_, Y is the dependent variable, X is the dietary Lys concentration, *β*_0_ is the value at the plateau, *β*_1_ is the slope and *β*_2_ is the Lys concentration at the break point.

^3^ Quadratic broken-line model (QBL): Y = *β*_0_ + *β*_1_ × (*β*_2_—X)^2^, where (*β*_2_—X) = 0 for X > *β*_2_, Y is the dependent variable, X is the dietary Lys concentration, *β*_0_ is the value at the plateau, *β*_1_ is the slope and *β*_2_ is the Lys concentration at the break point.

^4^ Exponential asymptotic (EA): Y = *β*_0_ + *β*_1_ × (1 –EXP(– *β*_2_ × (X– *β*_3_), where Y is the dependent variable, X is the dietary Lys concentration, *β*_0_ is the response for the dependent variable estimated for the feed with the lower Lys, *β*_1_ is the difference estimated between the minimum and maximum response obtained by the increasing Lys, *β*_2_ is the slope of the exponential curve, *β*_3_ is the Lys at the lower level; requirement were estimated by calculating (ln(0.05)/– *β*_2_) + *β*_3_ for 95% of the requirement and (ln(0.01)/– *β*_2_) + *β*_3_ for 99%.

^5^ Feed conversion ratio corrected for mortality weight.

## Discussion

Basal experimental diets utilized in the present research were based on corn, soybean meal, and corn gluten meal, and were formulated to contain CP and energy levels comparable to usual commercial diets in Brazil and US. It is crucial that requirement studies use a basal diet that is deficient in the tested AA to produce accurate estimates. Amino acid analysis determined that the basal diets in Exp. 1, 2, and 3 contained 1.12, 0.85, and 0.77% total Lys, respectively. Moreover, in these 3 experiments, broilers fed the basal diets presented poor performance compared with broilers fed the dose-response diets with increasing Lys levels, demonstrating that Lys was deficient in the basal diets.

In the present research, dig. Lys requirements varied depending upon the response criterion. Requirements for FCR was higher than BWG in Exp. 2 and 3, similar as observed in previous researches [12–14,16,29]. Apparently, as dig. Lys exceeds the concentration required for optimal BWG, growth rate is maintained while feed intake decreases, thus resulting in a higher requirement for FCR than BWG [3]. Furthermore, Lys increments produce more pronounced increases in BWG than feed intake and this is a consequence of FCR being a ratio between these variables. Therefore, as BWG increases, FCR is reduced thus increasing its requirement.

In parallel to results from the present study, previous research with broilers fed diets with progressive additions of dig. Lys had have shown quadratic increases in BWG and decreases in FCR in all studied ages [13,14,29,30]. Garcia and Batal (2005) [30] using the LBL model, estimated dig. Lys requirements ranging from 1.01 to 1.10% from 1 to 7 d, which is in agreement with our results from Exp. 1. Labadan et al. (2001) [12] estimated total Lys requirements from 1 to 14 d as 1.28% for BWG and 1.21% for FCR using the LBL model. Using the same model, Sklan and Noy (2003) [17] estimated lower dig. Lys requirements (0.92 to 0.96%) from 1 to 7 d. Digestible Lys requirements, from 1 to 14 d, using the QBL model was reported as 1.180% for BWG, which is in close agreement to our findings [14].

Dozier et al. (2009b) [16] estimated dig. Lys requirements from 14 to 28 d as 1.07% for BWG and 1.10% for FCR, respectively, using QP regression analysis, and as 1.09% for BWG and 1.15% for FCR, respectively, based on the QBL model. Although slightly lower, requirements from 12 to 28 d in Exp. 2 are similar to those previously reported. Furthermore, the QP regression demonstrated by Baker et al. (2002) [3] estimated dig. Lys requirements from 8 to 21 d as 0.97% for BWG and 1.03% for FCR using 90% of the maximal responses, which is in close agreement to the observed in Exp. 2. Labadan et al. (2001) [12] observed higher requirement estimate for BWG as 1.13% total Lys from 14 to 28 d using the LBL model.

Dozier et al. (2010) [13] estimated dig. Lys requirements from 28 to 42 d as 0.965% for BWG, 1.012% for FCR, 0.963% for carcass yield, and 0.981% for breast meat yield using QBL model, which are comparable to results in the present study. Similarly, Labadan et al. (2001) [12] using LBL model determined total Lys requirements as 0.94% for BWG and 0.95% for FCR. In contrast to our findings, Garcia et al. (2006) [18] estimated high dig. Lys requirements as 0.97, 0.96, 0.94, and 0.98% for BWG, FCR, and carcass yield, respectively, using the LBL model.

In the present study, 4 statistical models were generated in order to estimate dig. Lys requirements. Comparisons with previous research are valid; however, as breast muscles consistently increase as proportions of the total broiler due to genetic selection, the demand for Lys consumed also is expected to increase. Since genetic improvements also affect body weight gain and, therefore, feed intake, it could be questioned that the increased feed intake with the modern broiler would provide the increased Lys to sustain maximum breast yield. However, the breast muscle mass of the Ross 708 of 2009 broiler was 18% whereas the one from a heritage chicken of 1962 was 9% of the total body mass at 5 weeks [31]. Havenstein et al. (2003) [32] also concluded that genetics, nutrition and other management changes over the last 44 year have resulted in a 2001 broiler that required approximately one-third the time and over a threefold decrease in the amount of feed consumed to produce an 1,815 g Arbor Acres broiler. Additionally, Dozier et al. (2010) [13], using Cobb 700 broilers, also observed in two studies that the accelerated growth rate of broilers resulted in 10 to 12% higher digestible Lys requirements when expressed as a percentage of the diet than birds used in previous research.

The QP, LBL, QBL, and EA models have been thoroughly reviewed by Pesti et al. (2009) [15]. According to Pesti et al. (2009) [15], the QP regression analysis is simple to fit to data and maximum responses are easily obtained; however, it is not able to characterize the plateau that most nutritional responses are admitted to have. The broken-line models represent the concept of requirement, defined as the minimum nutrient level that result in maximum response; however more levels of the tested nutrient are required to obtain accurate estimates. Vedenov and Pesti (2008) [33] tested several nonlinear models, including LBL and QBL using different data sets and observed that one particular model is not necessarily the best for all nutritional responses and, therefore, the results of experiments should be the ones to dictate the model choice.

It is possible to compare models through their R^2^ and the sum of the square of residuals. Since the latter is a linear function of R^2^ values, the order of fit is the same. In the present research, all models tested were statistically significant (*P* ≤ 0.01). The model that provided the best fit was the QBL for BWG and FCR and the LBL for carcass and breast meat yield. The LBL model can underestimate requirements because response of broilers to increasing Lys levels is clearly nonlinear decreasing as the AA approaches its requirement what is popularly referred as the law of diminishing returns [25,27]. The QBL model provide a more accurate estimate of the Lys requirement when compared to the LBL model. The later simplifies the interpretation of growth curves if the response to a limiting AA is assumed to be sigmoidal [27]. On the other hand, the shape of QBL response curves does not allow for the safe limit for toxicity or growth impairment from a nutrient to be determined, which is possible with quadratic regression [15]. The model of choice, however, should meet both mathematical and biological aspects.

The comparisons between the present study with previous research has to take in consideration differences in studied variables such as strain, sex, age, type of diet, statistical model, and method (factorial vs. empirical). Overall, requirement estimates observed in the present research are notably higher than the recommended by the NRC (1994) [11]. The higher dig. Lys requirement observed in the present research was probably affected by genetics of the modern broiler, which allowed for less feed intake per unit of BW and greater meat accretion [13,32].

In conclusion, estimates of Lys requirements vary among response variables and statistical models; therefore, influencing recommendations to optimize economic performances. Throughout the 3 studies the requirement estimates for FCR were higher than BWG. Overall, the QBL model provided the best fit for growth performance variables and seemed adequate in both mathematical and biological backgrounds. Regardless of the model of choice, researchers should be familiar with the characteristics of each model to obtain precise and coherent requirements estimates.
